# Comparative Analysis of Testicular Transcriptional and Translational Landscapes in Yak and Cattle–Yak: Implications for Hybrid Male Sterility

**DOI:** 10.3390/biom15081080

**Published:** 2025-07-25

**Authors:** Mengli Cao, Shaoke Guo, Ziqiang Ding, Liyan Hu, Lin Xiong, Qianyun Ge, Jie Pei, Xian Guo

**Affiliations:** 1 Key Laboratory of Yak Breeding in Gansu Province, Lanzhou Institute of Husbandry and Pharmaceutical Sciences, Chinese Academy of Agricultural Sciences, Lanzhou 730050, China; 82101231285@caas.cn (M.C.); 82101221240@caas.cn (S.G.); 82101225482@caas.cn (Z.D.); 821012410407@caas.cn (L.H.); xionglin@caas.cn (L.X.); geqianyun@caas.cn (Q.G.); 2 Key Laboratory of Animal Genetics and Breeding on Tibetan Plateau, Ministry of Agriculture and Rural Affairs, Lanzhou 730050, China

**Keywords:** cattle–yak, testis, Ribosomal footprints, open reading frames, translation efficiency

## Abstract

Cattle–yak, a hybrid of yak and cattle, exhibits significant heterosis but male infertility, hindering heterosis fixation. Although extensive research has been conducted on transcriptional mechanisms in the testes of cattle–yak, the understanding of their translational landscape remains limited. In this study, we characterized the translational landscape of yak and cattle–yak based on Ribo-seq technology integrated with RNA-seq data. The results revealed that gene expression was not fully concordant between transcriptional and translational levels, whereas cattle–yak testes exhibited a stronger correlation across these two regulatory layers. Notably, genes that were differentially expressed at the translational level only (*MEIOB*, *MEI1*, and *SMC1B*) were mainly involved in meiosis. A total of 4,236 genes with different translation efficiencies (TEs) were identified, and the TEs of most of the genes gradually decreased as the mRNA expression level increased. Further research revealed that genes with higher TE had a shorter coding sequence (CDS) length, lower GC content, and higher normalized minimum free energy in the testes of yaks, but this characteristic was not found in cattle–yaks. We also identified upstream open reading frames (uORFs) in yak and cattle–yak testes, and the sequence characteristics of translated uORFs and untranslated uORFs were markedly different. In addition, we identified several short polypeptides that may play potential roles in spermatogenesis. In summary, our study uncovers distinct translational dysregulations in cattle–yak testes, particularly affecting meiosis, which provides novel insights into the mechanisms of spermatogenesis and male infertility in hybrids.

## 1. Introduction

Hybrid male sterility is a postzygotic reproductive isolation mechanism. In mammals, it refers to the infertility of heterogametic sex (male) offspring produced by interspecies hybridization due to their inability to generate functional gametes [1]. The cattle–yak (*Bos taurus* (*♂*) *× Bos grunniens* (*♀*)) combines the advantages of yaks, such as their adaptation to the high-cold and low-oxygen environment, ability to tolerate roughage, and strong disease resistance, with those of cattle, such as a fast growth rate and high meat production performance, and plays an increasingly important role in plateau animal husbandry [2,3,4]. However, male cattle–yaks are sterile due to abnormal spermatogenesis, which restricts the utilization of heterosis and has become the biggest obstacle to yak crossbreeding. Research shows that with the increase in backcross generations, cattle–yaks become completely fertile by the F_4_ generation, but their economic value is greatly reduced [1]. Elucidating the molecular mechanisms underlying hybrid reproductive barriers is crucial for enhancing the reproductive performance of hybrid species. Previous studies investigating the mechanisms underlying male infertility in cattle–yak have primarily focused on the transcriptional level. These studies revealed altered expression of genes associated with spermatogonial stem cell maintenance, cell differentiation, and meiosis in the cattle–yak [5,6,7,8,9]. However, the abundance of proteins is what ultimately determines the phenotype, and the translation level is the determinant of protein abundance [10]. The half-life, synthesis rate, and quantity of proteins and mRNA significantly differ. Changes in transcript levels can often be buffered or compensated for at the protein level [11,12]. However, the resolution of protein mass spectrometry is limited compared with nucleic acid sequencing methods [13]. Ribosome profiling sequencing (Ribo-seq) can resolve this dilemma. Ribo-seq can achieve single-codon-level resolution and obtain translation information on the genes, such as information on the ribosome distribution, starting codon position, programmatic frame shifts, and upstream open reading frames (uORFs) [14]. Ribo-seq can also be used to infer the instantaneous protein synthesis rate. Ribo-seq data combined with mRNA-seq data can be used to determine the translation efficiency (TE); potential functional micropeptides can also be identified [13].

The precision of translation regulation enables spermatogenic cells to carry out spermatogenesis normally; this allows sperm to undergo morphological and functional changes and finally mature into sperm capable of fertilization [15,16,17,18]. Previous studies have shown that translation is widely regulated in different organs, and differences in gene expression were approximately 20% lower at the translational level than at the transcriptional level, especially in the spermatogenic cells of the testis [19]. The up-regulation of translation compensates for the overall dose reduction during sex chromosome evolution and the effect of meiosis chromosome inactivation during spermatogenesis [19]. Therefore, merely analyzing mRNA and protein levels cannot provide sufficient information for a comprehensive understanding of gene expression. Here, we characterized differences in protein synthesis between yak and cattle–yak testes by combining Ribo-seq and RNA-seq. These results provide new insights that will aid analyses of male cattle–yak infertility.

## 2. Materials and Methods

### 2.1. Sample Collection

Three 4-year-old male yaks (Y: Y1, Y2, and Y3) and three male cattle–yaks (CY: CY1, CY2, and CY3; derived from a cross between Jersey cattle (♂) and Gannan yaks (♀)) from Linxia County, Linxia Hui Autonomous Prefecture (34°51′50′ N, 102°26′9′ E), in good physical condition and without any diseases, were sampled. Testicular tissues were collected after yaks were euthanized. The testicles of cattle–yak were obtained by castration via veterinary surgery. Collect the ipsilateral testicular tissues and immediately immerse them in liquid nitrogen for snap-freezing. The samples were sent to the Lanzhou Institute of Husbandry and Pharmaceutical Sciences of the Chinese Academy of Agriculture for subsequent Ribo-seq and Western blot (WB) to detect protein expression levels. Transcriptome data for the same sample have been uploaded to the National Center for Biotechnology Information (NCBI; https://www.ncbi.nlm.nih.gov; accession number: GSE208693 (accessed on 23 July 2022)) [5].

### 2.2. Ribosome Footprint (RF) Recovery, Library Construction, and Sequencing

Six samples were ground in liquid nitrogen and dissolved in 400 µL of lysis buffer. Ribosome analysis techniques were based on those in previously reported protocols [20]. The lysate was incubated at 4 °C for 10 min, and the cells were crushed 10 times through a 26 G needle. The lysates were centrifuged at 20,000× *g* for 10 min at 4 °C. Then, 10 µL of RNase I (NEB, Ipswich, MA, USA) and 6 µL of DNase I (NEB, Ipswich, MA, USA) were added to the supernatant and incubated at room temperature in a Nutator mixer (VWR, Radnor, PA, USA) for 45 min. Next, 10 µL of SUPERase In-RNase inhibitor (Ambion, Austin, TX, USA) was added to stop the digestion reaction. To recover ribosomes, 100 µL of digested RFs were loaded onto a pre-equilibrated size exclusion column (Illustra MicroSpin S-400 HR column, GE Healthcare, Chicago, IL, USA), centrifuged at 600× *g* for 2 min, and eluted with 10 µL of an eluent (*w*/*v*) containing 10% sodium dodecyl sulfate. Then, 10 µL of 10% (*w*/*v*) SDS was added to the elution, and RFs with a size greater than 17 nt were isolated using the RNA Clean and Concentrator-25 kit (Zymo Research, R1017, Irvine, CA, USA). rRNA was removed using a previously reported method [21]. RFs were further purified using magnet beads (Vazyme, Nanjing, China). Subsequently, the NEBNext^®^ Multiple Small RNA Library Prep Set for Illumina^®^ (catalog nos. E7300S and E7300L) was used to construct the Ribo-seq library, and sequencing was performed using the Illumina HiSeq^TM^X10 platform by Gene Denovo Biotechnology Co. (Guangzhou, China).

### 2.3. Data Filtering, Comparison, and Analysis of Ribosome Characteristics

Fastp (v0.20.0) was used to filter low-quality reads, including the removal of adapter-containing reads, reads with an N ratio greater than 10%, all A-base reads, and low-quality reads with a base number of Q ≤ 20 accounting for more than 50% of the entire read. Bowtie2 (v2.2.8) [22] was used to remove reads aligned to the rRNA. A BLAST (v2.11.0) search of the rRNA-removed reads was then conducted against the GenBank and Rfam databases to identify and remove tRNAs in the samples. Similarly, reads mapped to snoRNAs, snRNAs, and miRNAs were removed. Reads with a length of 20–40 bp were retained for subsequent analysis. STAR (v3.1.0) [23] was used to align the above data to the yak reference genome (Version: BosGru 3.0), and RFs were assigned to different genomic features according to the aligned 5′-end position. Three-nucleotide periodicity was plotted using the riboWaltz (v1.1.0) R package [24].

### 2.4. Gene Abundance Quantification and Differentially Translated Gene (DTG) Analysis

RSEM (v1.2.19) [25] was used to calculate the read count in the ORFs of the coding gene, and FPKM was used to normalize the gene expression levels. Correlation analysis was conducted in R, and principal component analysis (PCA) was conducted using the R package gmodels (v2.18.1.1). DESeq2 (v1.20.0) [26] was used to identify inter-group DTGs. Genes with a |log_2_ fold change (FC)| ≥ 1 and false discovery rate (FDR) < 0.05 in a comparison were considered significant DTGs. Gene Ontology (GO) and Kyoto Encyclopedia of Genes and Genomes (KEGG) pathway enrichment analyses were conducted. GSEA (v2.2.4) software [27] was used for gene set enrichment analysis.

### 2.5. Comparison of Transcriptional and Translational Differences

Pearson correlation coefficients (PCCs) between gene translation expression and transcript abundance in the group were calculated based on RNA-seq data [5], and a scatter plot was made. Based on the difference thresholds of genes in the two omics datasets, the genes were divided into the following five categories (based on the criteria |log_2_FC| ≥ 1 and FDR < 0.05): Transcription (only genes with significant transcriptional differences), Translation (only genes with significant translational differences), Homodirection (genes with significant differences in both omics datasets in the same directions), Opposite (genes with significant differences in both omics datasets but in opposite directions), and Unchanged (no significant differences in both omics datasets). After determining the numbers of various genes in each group, a scatter plot was made. The IGV browser was used to visualize the results at the whole-genome and single-gene levels. The STRING database was used to generate protein regulatory networks, and they were visualized in Cytoscape (Version 3.7.1, Paul Shannon, MA, USA).

### 2.6. TE (Translation Efficiency) Calculation and Analysis

TE was calculated using the following formula: TE = FPKM (Ribo-seq)/FPKM (RNA-seq) [28]. The coding sequence length, guanine–cytosine (GC) content, and normalized minimum free energy (NMFE) difference in the four TE groups (log_2_ (TE) ≤ −1, −1 < log_2_ (TE) ≤ 0, 0 < log_2_ (TE) ≤ 1, and log_2_ (TE) > 1) of yak and cattle–yak were calculated. RiboDiff [29] was used to identify differential translation efficiency genes (DTEGs) between groups; significant DTEGs were identified using the following criteria: |log_2_FC| ≥ 1 and FDR < 0.05. Based on the difference threshold of TE and the transcription level, the genes were divided into five categories (Transcription, TE, Homodirection, Opposite, and Unchanged; among these, TE represents genes that show significant differences only in translation efficiency) and differences in TE and transcription were compared.

### 2.7. uORF Identification and Analysis

We performed a customized ORFfinder search of transcript sequences annotated as non-coding regions and extracted untranslated region (UTR) sequences to identify uORFs containing AUG codons. Similarly, FPKM was used to normalize the translation levels of uORFs. The ORF score and ribosome release score (RRS) based on the abundance and location distribution of each uORF were calculated, and the Fickett score based on uORF sequence features and Hexamer score were also calculated. Potentially translated uORFs were determined based on the four aforementioned values. The sequence length and GC content of translated and untranslated uORFs (based on sequences with FPKM ≥ 1) in yak and cattle–yak were determined. NMFE was used to define the sequence stability of secondary structures, and this was calculated by ViennaRNA RNAfold (v2.1.9) [30] and normalized by the sequence length. *p*-values were determined by Student’s *t*-test between translated and untranslated uORFs. Motif analysis of the starting codons of all uORFs was performed using MEME, and significance analysis was conducted using chi-square tests. The Kozak sequence of uORFs and the effect of the distance between uORFs and major ORFs (mORFs) on the translation ability were determined for both translated and untranslated uORFs. A scatter plot of differential changes between uORFs and downstream mORFs was made. Correlations between the uORF length, GC content, NMFE, and TE of the gene were calculated, and the regression diagram was made using the R package visreg.

### 2.8. Protein Extraction and Determination

Six samples were ground and weighed in liquid nitrogen, and 50 mg was dissolved in 400 µL of high-efficiency RIPA tissue lysate (R0010, Solarbio, Beijing, China) of phenylmethanesulfonyl fluoride (1 mM, P0100-1, Solarbio) [31]. After three cycles of freezing and thawing, centrifugation was performed at 12,000× *g* for 15 min, and the supernatant was collected. After the replicate samples in the group were fully mixed, the BCA protein detection kit (PC0020, Solarbio) was used to determine the protein concentration. The sample protein concentration was diluted to 5 µg/µL using RIPA lysis. Protein samples were isolated using 4–20% ProteinEle Precast Tris–Glycine Gel (PG42010-N, Solarbio). The concentrated gel was cut based on the size of the target protein band and transferred to a polyvinylidene fluoride membrane (PVDF, 0.2 µm, ISEQ00010, Immobilon, Darmstadt, Germany). The PVDF membrane was blocked in protein-free rapid blocking solution (SW3012, Solarbio) for 1.5 h and incubated with primary antibody at 4 °C overnight. The rabbit polyclonal antibodies used included MEI1 (1:500, bs-6387R, Bioss, Beijing, China) and FTSJ1 (1:1500, 11620-1-AP, Proteintech, Wuhan, China), and the mouse polyclonal antibody used was β-actin (1:4000, 66009-1-AP, Proteintech). After incubation, the membrane was washed with TBST solution (Tris-buffered saline containing 0.1% Tween-20); it was then incubated with horseradish peroxidase-conjugated goat anti-rabbit lgG (1:4000, HS101-01, Transgen, Beijing, China) and goat anti-mouse lgG (1:4000, HS201-01, Transgen) at 37 °C for 1 h. Protein bands were visualized by adding WesternBright ECL HRP substrate (K-12045-D50, Advansta, California, CA, USA). Protein images were obtained using a chemiluminescence instrument. The final measurements were taken and calculations were performed using ImageJ (v1.54p) software. Statistical analysis was performed using SPSS 26.0 (IBM, Armonk, NY, USA). After assessing the homogeneity of variance and normality of the data using the Shapiro–Wilk test and Levene’s test, the independent-samples *t*-test was employed for analysis.

## 3. Results

### 3.1. Read Quality, Alignment, and Ribosome Footprint (RF) Distribution According to Testicular Ribo-Seq

**Figure 1:**
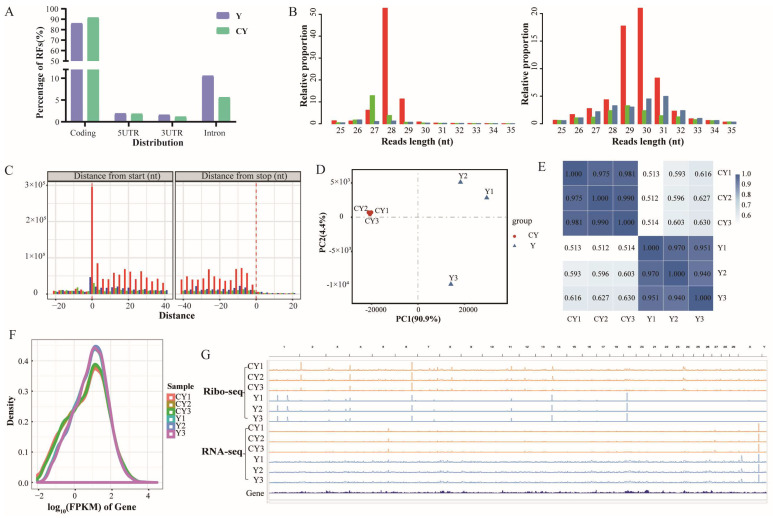
RNA-seq and Ribo-seq data of yak testes. (**A**) Distribution of RFs for CY and Y; (**B**) CY (**left**) and Y (**right**) RF alignment codon distribution map. (**C**) Distribution of trinucleotides. (**D**) PCA plot of samples. (**E**) Sample correlation heatmap. (**F**) Log_10_FPKM distribution of Ribo-seq data. (**G**) IGV browser view showing global RNA-seq and Ribo-seq landscape of yak and cattle–yak testicles.

A total of 99,624,864 and 119,345,057 clean reads were obtained on average in the yak and cattle–yak groups, respectively (Supplementary Table S1). Reads mapped to rRNAs, tRNAs, snRNAs, snoRNAs, and miRNAs were removed (Supplementary Table S2). Given that the ribosome-protected fragments were approximately 30 bp, we only retained reads with a length of 20 bp to 40 bp for subsequent analysis. The obtained reads were aligned to the yak reference genome sequence (version: BosGru 3.0) using STAR (v3.1.0) software, and RFs were obtained; the comparison rate was approximately 95.51% (cattle–yak) and 79.88% (yak) (Supplementary Table S3). Most RFs were mapped to the coding sequence (CDS) region of coding genes (yak: 86.03%; cattle–yak: 91.47%); RFs were rarely mapped to the UTR (Figure 1A).

The main peaks in the lengths of RFs in cattle–yak and yak were 28 nt and 30 nt, respectively (Figure 1B). Given that the residence time of the ribosome is longest at the first base position of the codon, the RFs in the CDS region showed a clear ‘high–low–low’ distribution of trinucleotides (Figure 1C). The yak and cattle–yak groups could be clearly distinguished in a PCA plot (Figure 1D), and the PCC of repeated samples in the group was greater than 0.94 (Figure 1E). This high correlation is consistent with the expression distribution of Ribo-seq data (Figure 1F). These findings demonstrate the reliability and accuracy of the Ribo-seq data. The global transcriptional and translational landscapes of yak and cattle testes are presented in Figure 1G.

### 3.2. Translational Down-Regulation of Spermatogenesis Genes Is Prevalent in Cattle–YAK Testis

**Figure 2:**
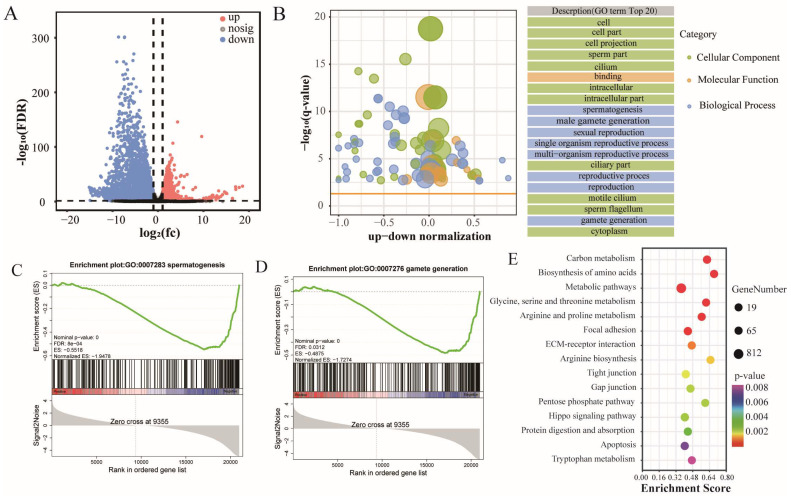
The difference in gene translation levels between CY and Y testes. (**A**) DTG volcano map between CY and Y. (**B**) GO enrichment bubble plot of CY and Y DTGs. GSEA of spermatogenesis (**C**) and gamete generation (**D**) gene sets showing the normalized enrichment score (FDR, false discovery rate; positive and negative normalized enrichment scores indicate higher and lower expression in CY, respectively). (**E**) KEGG enrichment bubble plots of CY and Y DTGs.

The number of RF genes detected in the cattle–yak and yak groups was 18,918 and 19,115, respectively. We identified 2,760 and 4,734 translationally up-regulated and down-regulated genes in cattle–yak, respectively, using the following criteria: FDR < 0.05 and |log_2_FC| > 1 (Supplementary Table S4 and Figure 2A). The top five genes in DTGs include *ACE*, *GAPDHS*, and *PDGFRA*. GO enrichment analysis showed that DTGs were involved in spermatogenesis, gamete generation, sperm part, cilium, motile cilium, and sperm flagellum (Figure 2B). Gene set enrichment analysis (GSEA) revealed that the translation levels of spermatogenesis (Figure 2C) and gamete generation (Figure 2D) gene sets were generally reduced in cattle–yak. The translation level of genes involved in sperm part, cilium, motile cilium, and sperm flagellum was generally lower in cattle–yak than in yak (Supplementary Figure S1). Kyoto Encyclopedia of Genes and Genomes (KEGG) enrichment analysis showed that DTGs were mainly enriched in Focal adhesion, ECM–receptor interaction, Tight junction, Hippo signaling pathway, and Apoptosis and other pathways (Figure 2E). These pathways play an important role in cell migration, proliferation, differentiation, apoptosis, and testicular spermatogenesis.

### 3.3. Transcription–Translation Coordination Controls Spermatogenesis

**Figure 3:**
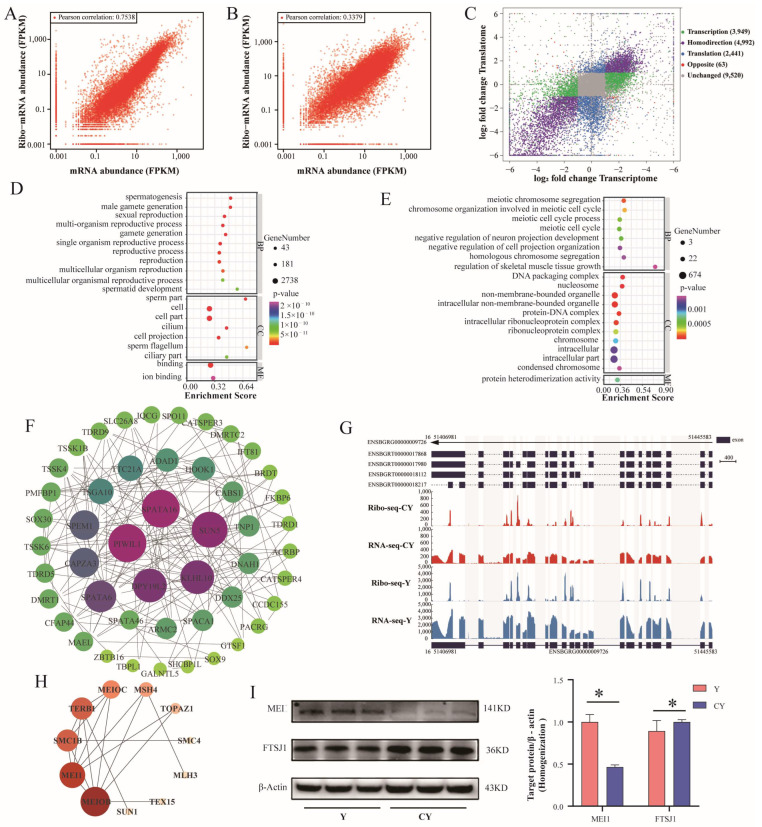
Comparison of transcription and translation levels between yak and cattle–yak testes. Correlation analysis of translational and transcriptional expression levels between cattle–yak (**A**) and yak (**B**). (**C**) Comparison and classification chart of translational and transcriptional differences. GO enrichment analysis of genes in the Homodirection (**D**) and Translation (**E**) groups. (**F**) PPI network diagram of genes involved in spermatogenesis. (**G**) RNA-seq and Ribo-seq IGV visualization of PIWIL1. (**H**) PPI network diagram of meiosis-related genes, including those involved in meiotic chromosome segregation, chromosome organization involved in meiotic cell cycle, meiotic cell cycle process, and meiotic cell cycle. (**I**) WB detection of the expression levels of MEI1 and FTSJ1 (* represents *p* < 0.05). Original figures can be found in Supplementary Materials.

We found that the translation and transcription levels were positively correlated in cattle–yak and yak, but this correlation was stronger in cattle–yak (Figure 3A) than in yak (Figure 3B). The proportions of genes in five categories (based on the criteria |log_2_FC| ≥ 1 and FDR < 0.05) characterized by different magnitudes and directions of translation and transcription were as follows (Figure 3C and Supplementary Table S5): Transcription (18.84%, 3949), Translation (11.64%, 2441), Homodirection (23.81%, 4992), Opposite (0.30%, 63), and Unchanged (45.41%, 9520).

We conducted GO enrichment analysis on genes in the Homodirection and Translation groups. Homodirection-group genes were enriched in spermatogenesis, male gamete generation, sexual reproduction, and spermatid development (Figure 3D). Translation-group genes were enriched in biological processes related to meiosis, such as meiotic chromosome segregation, chromosome organization involved in meiotic cell cycle, meiotic cell cycle process, and meiotic cell cycle (Figure 3E). We performed PPI analysis on 135 genes enriched in spermatogenesis in the Homodirection group and retained genes with a Degree > 3 (Figure 3F); the top five most connected genes were *SPTAT16*, *PIWIL1*, *DPY19L2*, *KLHL10*, and *SUN5*. The IGV view showed that the transcription and translation levels of *PIWIL1* were significantly reduced in the testes of cattle–yak (Figure 3G). PPI analysis was performed on the genes involved in meiosis in the Translation group. The three genes with the most connections were *MEIOB*, *MEI1*, and *SMC1B* (Figure 3H). The WB results showed that the expression levels of *FTSJ1* (Opposite) and *MEI1* (Translation) were significantly increased and decreased in the testes of cattle–yak, respectively (Figure 3I), which was consistent with the results of Ribo-seq.

### 3.4. Identification of DTEGs and Analysis of CDS Characteristics

**Figure 4:**
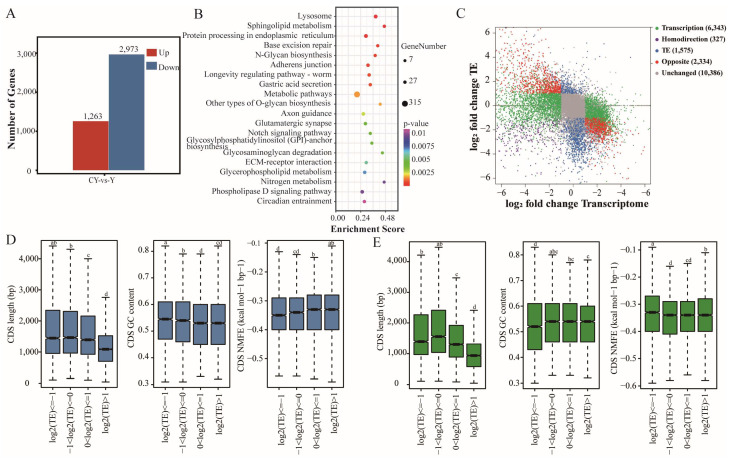
Differences in TE between CY and Y testicular genes. (**A**) Statistics of DTEGs for yak and cattle–yak. (**B**) KEGG enrichment bubble plot of DTEGs. (**C**) Comparison and classification chart of TE differences and transcriptional differences for genes between yak and cattle–yak. Differences in CDS length, GC content, and normalized minimum free energy (NMFE) among the four TE groups of yak (**D**) and cattle–yak (**E**), with significant differences represented by different letters (*p* < 0.05).

TE can directly measure the utilization efficiency of RNA, which is calculated as FPKM (Ribo-seq)/FPKM (RNA-seq). A total of 4,236 DTEGs were identified in yak and cattle–yak (Figure 4A and Supplementary Table S6), of which 1,263 were up-regulated and 2,973 were down-regulated in cattle–yak. According to the KEGG analysis, the DTEGs were mainly enriched in metabolic pathways, lysosome, ECM–receptor interaction, and Notch signaling pathway (Figure 4B).

The genes were divided into five categories based on differences between the TE and transcription level (Supplementary Table S7). The number of genes in the Opposite group (2,334 genes) was much higher than that in the Homodirection group (327 genes) (Figure 4C), indicating that the TE of most genes gradually decreases as the mRNA expression level increases. We also analyzed the effect of CDS characteristics on the translation of genes in yak and cattle–yak testes. We found that genes with higher TE in yak testes have shorter CDS lengths, lower GC content, and higher normalized minimum free energy (NMEF) (Figure 4D). However, the TE level of genes in the testes of cattle–yak was not consistent with the aforementioned sequence characteristics of the CDS region (Figure 4E).

### 3.5. Characteristics of uORFs and Their Effect on mORF Translation

**Figure 5:**
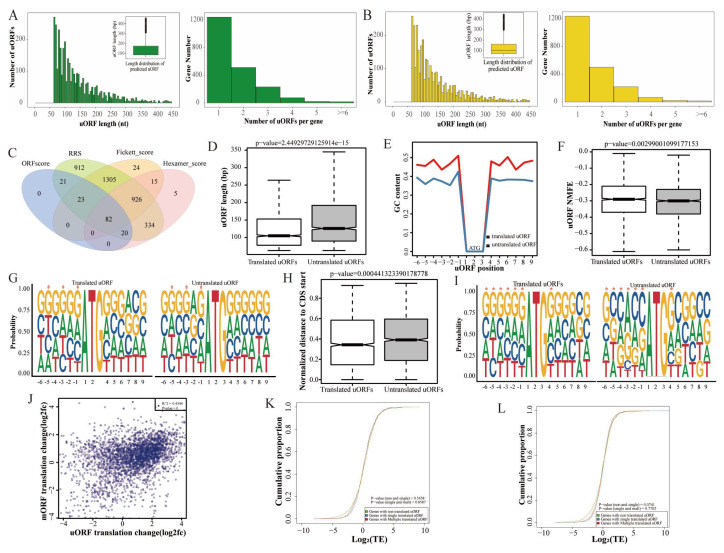
uORF features. Distribution of the number and length of uORFs in yak (**A**) and cattle–yak (**B**). (**C**) Potentially translated uORF screening. Comparison of the length (**D**), GC content (**E**), NMFE (**F**), Kozak sequence (**G**), and distance to the start of the CDS from (**H**) translated and untranslated uORFs in yak. (**I**) Comparison of Kozak sequences of yak uORFs and mORFs (* represents *p* < 0.05). (**J**) Scatter plot of differential changes in yak uORFs and downstream mORFs. (**K**) Cumulative curve of the effect of different types of uORFs on TE in yaks (**K**) and cattle–yaks (**L**).

uORFs are ORFs whose initiation codons are positioned upstream of the CDS, and they can inhibit the translation initiation of CDSs by competitively binding to ribosomes. We provide a more detailed description of the uORFs in yak and cattle–yak. There were 3,499 uORFs in 2,012 genes in yak (Figure 5A) and 3,380 uORFs in 2,061 genes in cattle–yak (Figure 5B). The length of the uORFs mostly ranged from 50 to 200 bp, and more than half of the genes had only one uORF. We predicted the coding ability of all identified uORFs based on analysis of the ORF score, RRS, Fickett score, and Hexamer score. A total of 82 potentially translated uORFs were screened (Figure 5C). We annotated the identified uORFs with an average FPKM ≥ 1, and uORFs in six genes were annotated, including *EIF4G1*, *CAMTA2*, and *PKP4* (Supplementary Table S8).

We then characterized translated and untranslated uORF sequences of yak and cattle–yak, and the length of the translated uORF sequences was shorter than that of untranslated uORF sequences (Figure 5D and Supplementary Figure S2A); in addition, the GC content was higher in the translated uORF sequences than in untranslated uORF sequences (Figure 5E and Supplementary Figure S2B). The NMFE of translated uORFs was higher than that of untranslated uORFs in yak (Figure 5F); no significant difference in the NMFE of translated and untranslated uORFs was observed in cattle–yak (Supplementary Figure S2C). Kozak sequence analysis of uORFs showed that the Kozak sequence of translated uORFs was more conserved than that of untranslated uORFs. For example, the occurrence probability of cytosine at the translated uORF-5 position of yak and cattle–yak was higher than that of untranslated uORFs (Figure 5G and Supplementary Figure S2D). The distance between the last uORF of each gene and the mORF start codon (CDS start) was calculated. The normalized distance from the translated uORF to the CDS start of the mORF is shorter in both yak (Figure 5H) and cattle–yak (Supplementary Figure S2E). The Kozak sequence of mORFs was more conserved than that of uORFs (Figure 5I).

To clarify the effect of uORFs on the translation of downstream mORFs, we made a scatter plot of differential changes in uORFs and downstream mORFs with FPKM ≥ 1 in at least one group (Figure 5J), which showed moderate correlation. In our study, the number of untranslated uORFs and translated uORFs had no significant effect on the TE of genes in yak (Figure 5K) and cattle–yak (Figure 5L).

## 4. Discussion

**Figure 6:**
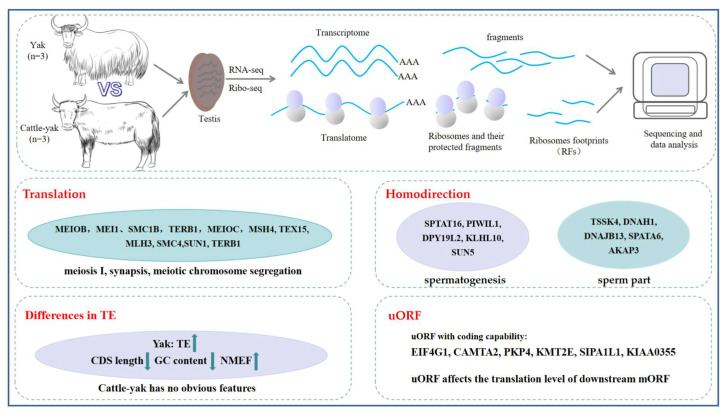
Schematic of Ribo-seq and RNA-seq principles and results for yak and cattle–yak testicular tissues.

Several studies have characterized changes in the mRNA levels of genes involved in the arrest of spermatogenesis in cattle–yak [7]. However, the expression of protein-coding genes might be regulated at the post-transcriptional level. The role of the translation level in testicular spermatogenesis arrest in cattle–yak has not yet been studied. Through integrated Ribo-seq and RNA-seq analyses, we found that spermatogenesis genes exhibit not only transcriptional defects but also widespread translational suppression. Specifically, the dysregulation of meiotic regulators, structural components of sperm part, and uORF-mediated translational control mechanisms elucidates novel pathogenic pathways (Figure 6). Our findings provide new insights with implications for the study of spermatogenesis arrest in cattle–yak, and the specific mechanism of action needs further investigation.

The RFs of both yak and cattle–yak in the CDS region showed a clear ‘high–low–low’ distribution of trinucleotides, which was consistent with the findings of a previous study [32]. The main peak in the RF length was between 28 and 30 nt, which is consistent with that observed in mammals [33]. The abundance of *ACE* and *GAPDHS* was significantly lower in cattle–yak testes than in yak testes. High levels of testicular ACE are only present in round and elongated sperm cells. This specificity stems from an intragenic promoter that is only active in male germ cells [34], which is also consistent with the presence of only a small number of round sperm cells and the lack of long sperm cells in cattle–yak [6]. *PDGFRA* is a marker gene of stem Leydig cells, and it was more highly expressed in cattle–yak than in yak [35]. The expression of the Leydig cell marker genes *ACTA2*, *IGF1*, *MYH11*, and *NR4A1* was also significantly increased, indicating that new Leydig cells could be regenerated in cattle–yak; this might also explain why cattle–yak could secrete testosterone normally and had normal sexual desire [36]. GSEA showed significant translational down-regulation of spermatogenesis and sperm composition genes in cattle–yak testes versus yaks. This is consistent with the general speculation that spermatogenesis arrest in cattle–yak begins when spermatogonial stem cells differentiate into spermatocytes and intensifies during meiosis [37]. The KEGG results showed that DTGs were enriched in pathways such as Focal adhesion, ECM–receptor interaction, and Tight junction. The ECM–receptor signaling pathway can regulate cell proliferation and differentiation, which affects the differentiation process from spermatogonia to spermatocytes [38]. Focal adhesion proteins are also critically important for ensuring normal meiosis [39]. Tight junctions are a major component of the blood–testis barrier, which helps maintain the microenvironment required for normal sperm production [40]. The dysregulated expression of genes related to these pathways in cattle–yak may contribute to the stagnation of their sperm.

Accurate spermatogenesis is precisely regulated at the transcriptional, post-transcriptional, and translational levels [41]. We conducted a joint analysis of the transcriptome and translation patterns of yak and cattle–yak testes. We found that the PCCs between the transcription and translation of genes were higher in cattle–yak than in yak. This may stem from the lack of spermatogenic cells of different types in the testis of cattle–yak, which affects post-transcriptional regulation. The landscape of testicular transcription and translation levels is not consistent. We found that the translational expression levels of certain key meiosis-related genes (such as *Mei1*, *MEIOB*, and *SMC1B*) in cattle–yak testes were significantly lower than those in yak, with no significant differences observed at the transcriptional level. This decoupling of transcriptional and translational regulation may reflect the involvement of post-transcriptional mechanisms. Previous studies have demonstrated that m6A modification can affect mRNA stability, and nuclear and translation efficiency [42], and there may be abnormal m6A modification in cattle–yak. RNA-binding proteins (RBPs) can modulate translation efficiency by interacting with 5′UTR or 3′UTR regions without altering mRNA abundance [43]. Additionally, activation of specific signaling pathways, such as mTOR, may selectively enhance the assembly of translation initiation complexes to promote translation of target mRNAs [44]. Mei1 is necessary for homologous recombination during meiosis in mammalian spermatogenesis [45,46]. The methylation levels of the *Mei1* promoter and genome are significantly higher in yak than in cattle–yak [47], and our results indicate that the Mei1 protein level was significantly lower in cattle–yak testes than in yak testes. *MEIOB* plays a critical role in the recruitment of recombinases during the early stages of homologous recombination and in the formation of crossovers in the mid-to-late phases of recombination. Mutations at multiple sites in *MEIOB* are associated with male infertility [48,49]. SMC1B, a specific component of the meiotic cohesin complex, is essential for meiotic chromosome dynamics, DNA recombination, axial element formation, and maintenance of telomere integrity [50]. The PIWIL1 protein can bind to piRNAs (PIWI-interacting RNAs), which affects the post-transcriptional regulation of gene expression. Spermatogenesis during meiosis I is severely impaired in PIWIL1-deficient golden hamsters [51]. In our study, *PIWIL1* in cattle–yak was significantly lower in both transcription and translation levels compared to yak, which is consistent with the results of Gu et al. [52].

The study of TE can provide insights into rapid changes in cell life activities. Our findings demonstrate that the TE of genes was higher in yak testes than in cattle–yak testes, which might stem from the fact that the complete spermatogenesis process requires the efficient synergy of multiple proteins. The KEGG results showed that lysosomes comprised the most significant signaling pathway for DTEGs, and 53 genes were enriched in this pathway; the TE of 49 genes was lower in cattle–yak than in yak. However, lysosomes are degradation centers and signaling hubs in cells, and they play key roles in maintaining cellular homeostasis [53]. Autophagy based on lysosomes is necessary for normal cell survival and plays a key role in the normal development of testicular cells [54]. Our findings indicate that as the mRNA expression level of most genes increases, TE gradually decreases, which is consistent with the results of a previous study of mammalian testes [19]. This might stem from mRNA modification, mRNA alternative splicing, and miRNA-mediated silencing or negative feedback inhibition associated with the translation of corresponding genes when the protein level increases. The sequence characteristics of genes affect increases in the proportion of mRNAs associated with ribosomes, as well as protein abundance [55]. High-TE genes in yak have shorter CDSs, a lower GC content, and higher NMEF than low-TE genes, which was consistent with the results of previous studies [56]. Shorter CDSs can reduce the probability of premature ribosome drop-off during translation [57]. Meanwhile, we speculate that in the high-altitude hypoxic environment, where energy metabolism is constrained, the low GC content and high NMEF weaken the stability of mRNA secondary structures, thereby adapting to the demands of translation kinetics under low-temperature conditions [58]. Meanwhile, the mRNA degradation rate may be suppressed by local regulatory factors (such as piRNA and miRNA) [59]. The absence of these characteristics in cattle–yak may prevent critical genes involved in spermatogenesis from synthesizing sufficient protein correctly. In our study, over half of the genes only had one uORF, but some genes contained up to thirteen uORFs. Six coding proteins were successfully annotated based on the identified uORFs. This is consistent with the results of a previous study showing substantial variation in the number of uORFs carried by each gene, and the number of uORFs that can be successfully annotated was less than 1% of the total number of uORFs [60]. EIF4G1 is a coding protein involved in the initiation of eukaryotic translation. It recruits mRNA to the translation machine by binding to EIF4E, thereby initiating protein synthesis, which is essential for the synthesis of a large number of proteins in spermatogenesis [61]. In our study, the translation level of *EIF4G1* in cattle–yak testes was significantly down-regulated, which may lead to specific mRNA translation failures. The flanking sequence of the translated uORF was more conserved than the Kozak sequence compared with the untranslated uORF, which was also observed in a mammalian model [62]. The Kozak sequence is one of the main factors affecting initial codon recognition [63]. When the AUG is located in the non-ideal Kozak sequence, the uORF coding level is low [64]. uORF is a *cis*-element that plays a widespread role in mORF translation inhibition, and it can inhibit the translation initiation of CDSs by competitively binding to ribosomes [65]. In our study, the number of untranslated uORFs and translated uORFs had no significant effect on gene TEs, which is inconsistent with the findings of many studies [66,67]. An ATG start codon in a poor context or a near-cognate start codon will increase the rates of leaky scanning, thereby decreasing the inhibitory effect of the uORF [68]. In our study, the Kozak sequence of the mORFs was more ideal than that of the uORFs, which might result in the disappearance of this inhibitory relationship. Although this study preliminarily revealed the critical role of translational regulation in spermatogenesis arrest in cattle–yak, the impact of uORFs on target-mORF TE is highly complex, and their precise mechanisms require further exploration. Meanwhile, this study used only three biological replicates. Although our PCA and correlation analyses confirmed the reliability of the data, future research will benefit from additional biological replicates to uncover more comprehensive and subtle effects.

## 5. Conclusions

In summary, we constructed a translation landscape of yak and cattle–yak testes, integrated transcriptome data, and identified key genes such as those encoding PIWIL1 and MEI1, suggesting that spermatogenesis is co-regulated by transcription and translation. A large number of uORFs were identified, and these encoded a small number of proteins and regulated the expression of downstream coding genes. These findings provide new insights with implications for the study of testicular spermatogenesis and male sterility in cattle–yak.

## Data Availability

The Ribo-seq dataset in this study can be found in GEO (accession number: GSE270057 (Accessed on 30 August 2024)).

## Supplementary Materials

The following supporting information can be downloaded at: https://www.mdpi.com/article/10.3390/biom15081080/s1, Supplementary Figure S1. GSEA of sperm part (A), cilium (B), motile cilium (C), and sperm flagellum (D) gene sets; normalized enrichment score. FDR, false discovery rate. Positive and negative normalized ES indicate higher and lower expression in CY. Supplementary Figure S2. uORF characteristics of cattle–yak. The length (A), GC content (B), NMFE (C), Kozak sequence (D), and distance to the mORF (E) of translated uORFs and untranslated uORFs in cattle–yaks were compared. Supplementary Table S1. Ribo-seq data read filtering information statistics. Supplementary Table S2. Comparison of rRNA/tRNA/snoRNA/snRNA/miRNA statistics. Supplementary Table S3. Ribo-seq alignment gene statistics. Supplementary Table S4. DTG statistics. Supplementary Table S5. Statistical analysis of genes classified based on differences in the direction and magnitude of translation and transcription. Supplementary Table S6. Statistics of genes showing differences in TE between yak and cattle–yak. Supplementary Table S7. Statistics of genes in groups varying in the direction and magnitude of TE and transcription. Supplementary Table S8. uORF annotation. All Western Blot original images can be found in the Supplementary Materials.
