# Supplementary figures and images for: Comparative Analysis of Testicular Transcriptional and Translational Landscapes in Yak and Cattle–Yak: Implications for Hybrid Male Sterility
Source: Biomolecules. 2025 Jul 25;15(8):1080. doi: 10.3390/biom15081080 (PMC12383912; doi:10.3390/biom15081080)

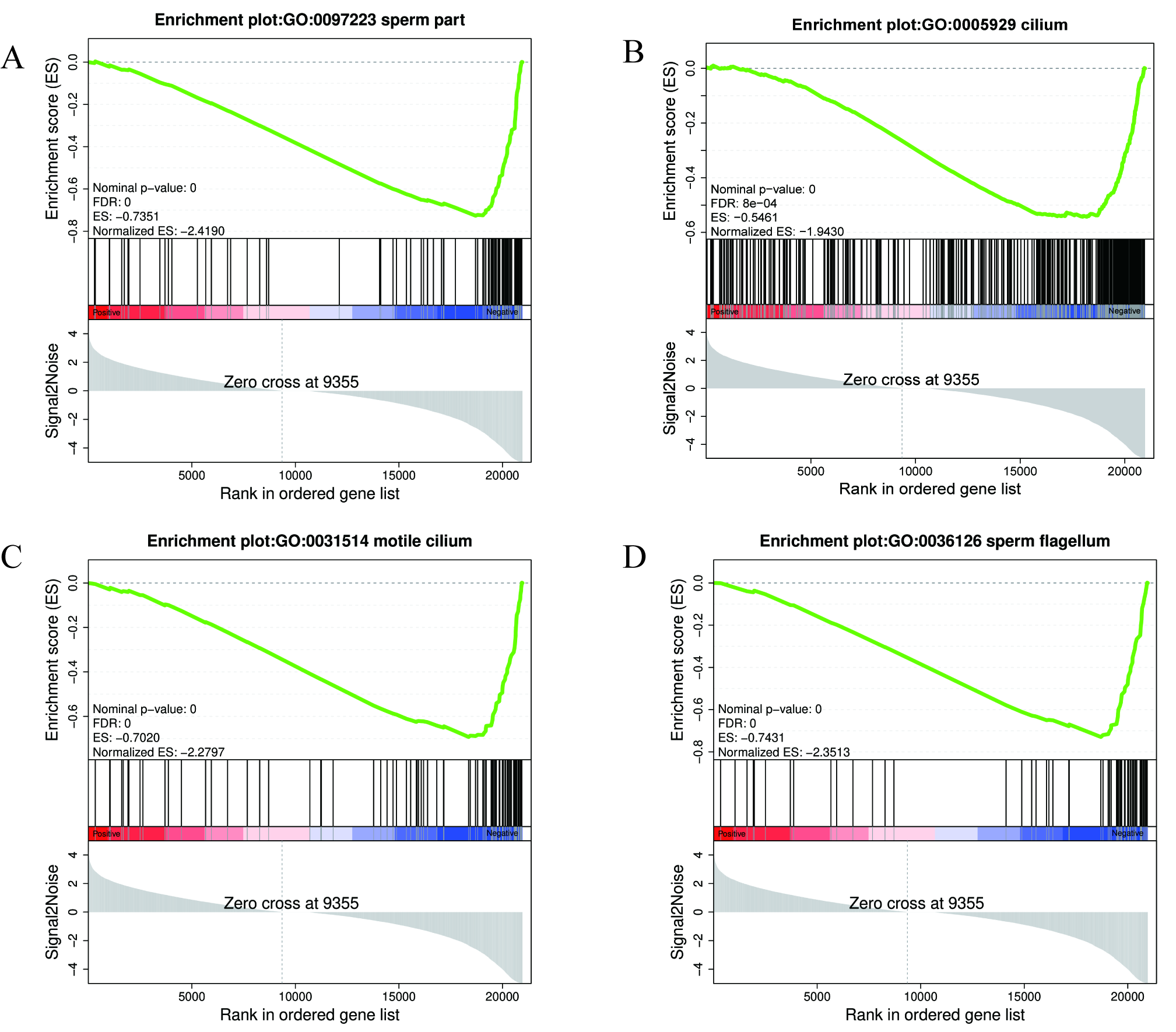

Supplement: Supplementary file 1 [file biomolecules-15-01080-s001.zip › biomolecules-3675083-Supplementary File/biomolecules-3675083-Supplementary/Supplementary Figure 1.tif]

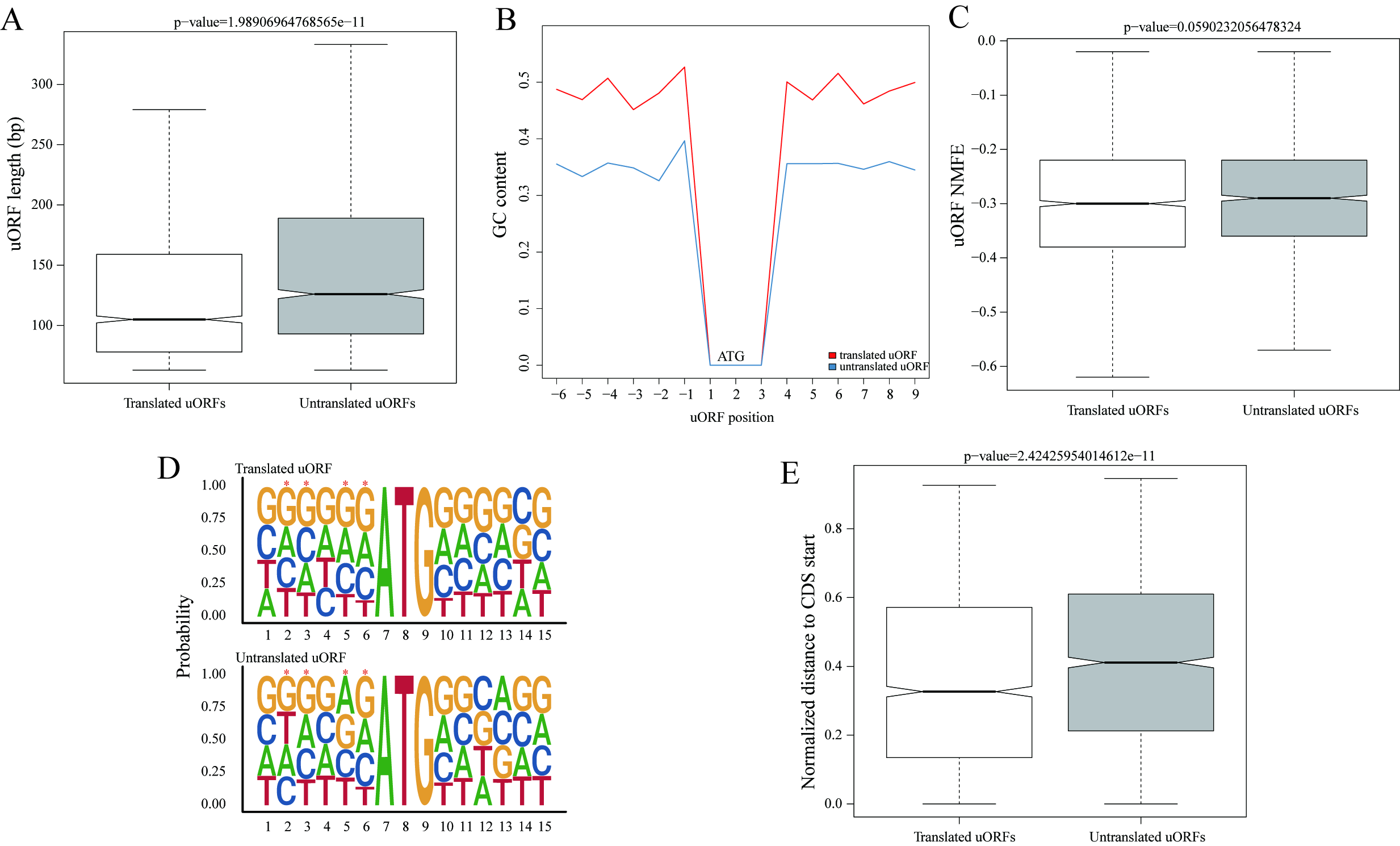

Supplement: Supplementary file 1 [file biomolecules-15-01080-s001.zip › biomolecules-3675083-Supplementary File/biomolecules-3675083-Supplementary/Supplementary Figure 2.tif]

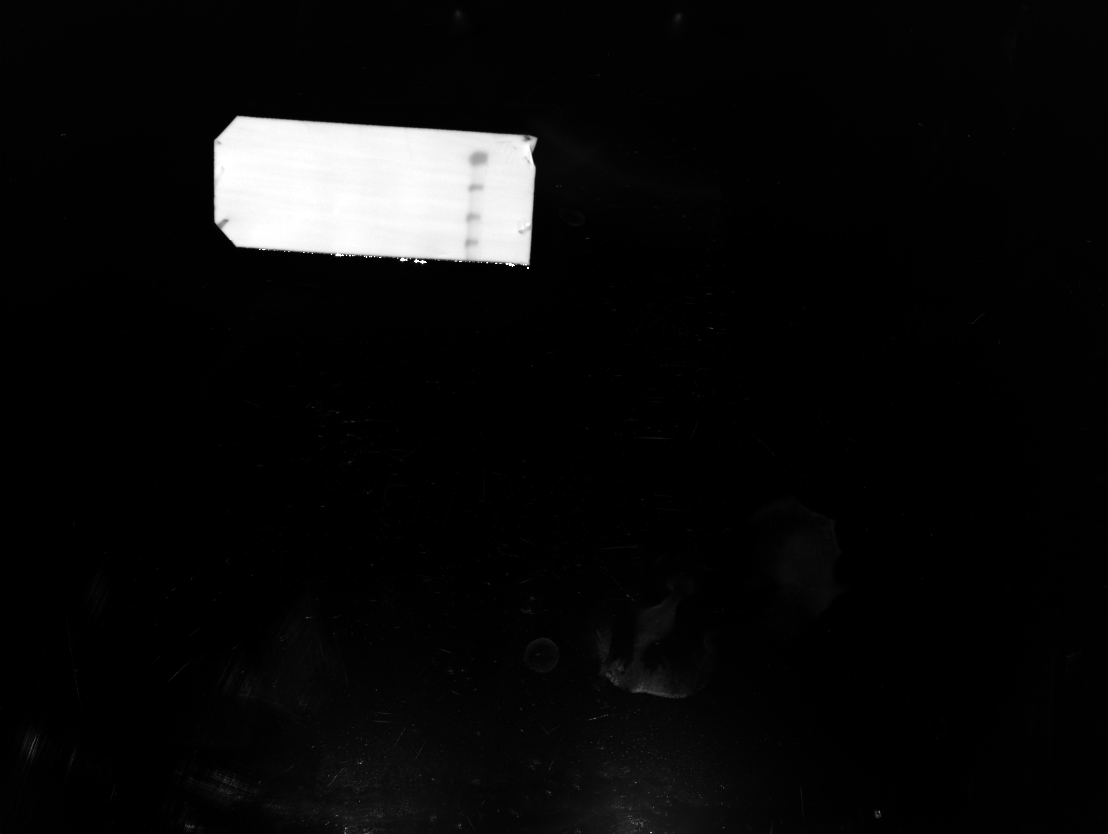

Supplement: Supplementary file 1 [file biomolecules-15-01080-s001.zip › biomolecules-3675083-Supplementary File/biomolecules-3675083-Western blots/Western blots/FTSJ1 Marker.png]

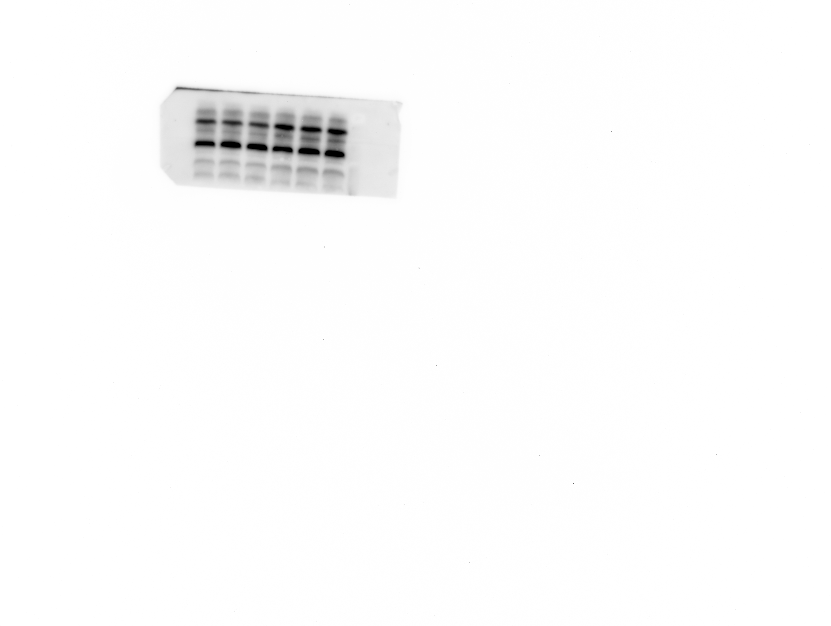

Supplement: Supplementary file 1 [file biomolecules-15-01080-s001.zip › biomolecules-3675083-Supplementary File/biomolecules-3675083-Western blots/Western blots/FTSJ1.png]

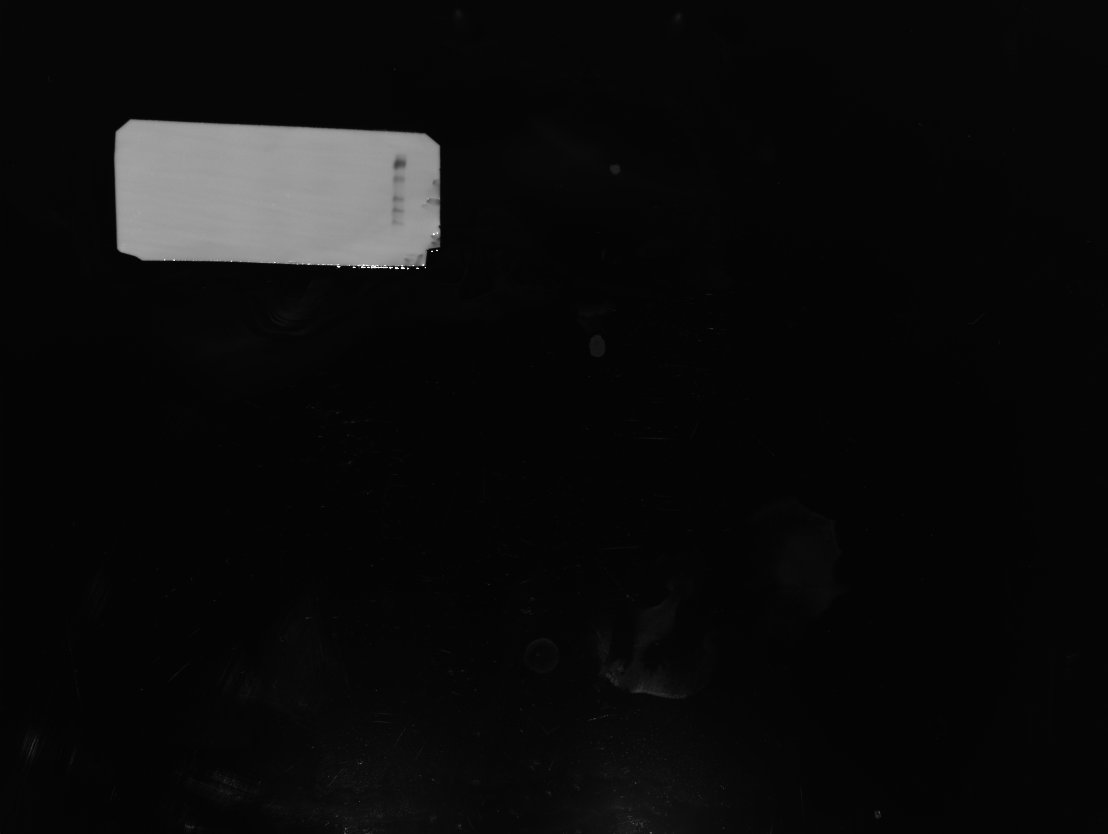

Supplement: Supplementary file 1 [file biomolecules-15-01080-s001.zip › biomolecules-3675083-Supplementary File/biomolecules-3675083-Western blots/Western blots/MEI1 Marker.png]

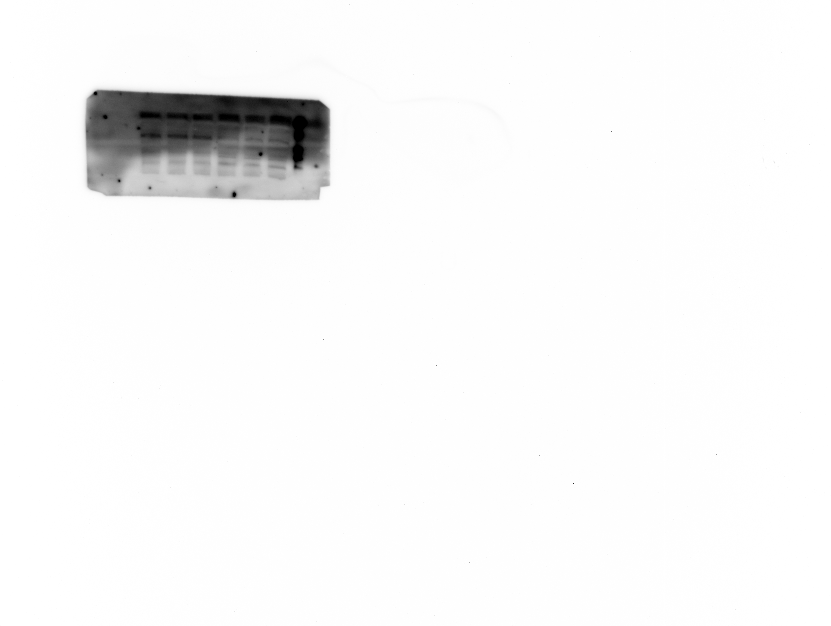

Supplement: Supplementary file 1 [file biomolecules-15-01080-s001.zip › biomolecules-3675083-Supplementary File/biomolecules-3675083-Western blots/Western blots/MEI1.png]

$\beta$ -Actin

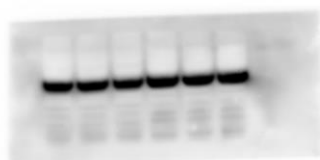

43KD

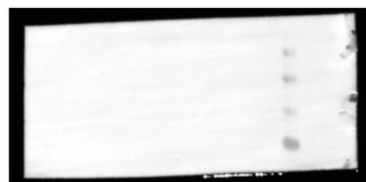

63KD

48KD

35KD

25KD

Marker

FTSJ1

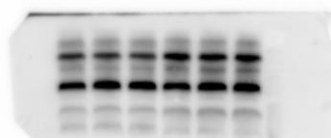

36KD

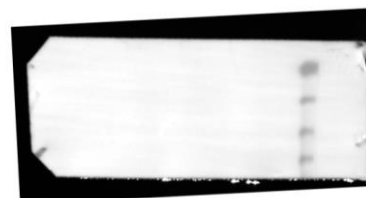

25KD

35KD

48KD

63KD

Marker

MEI1

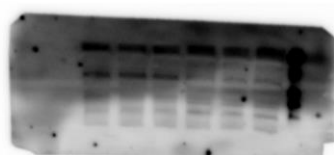

141KD

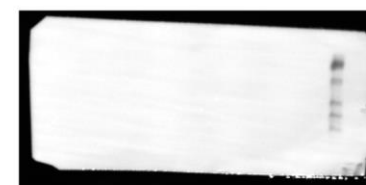

75KD

100KD

135KD

180KD

245KD

Marker

Supplement: Supplementary file 1 [file biomolecules-15-01080-s001.zip › biomolecules-3675083-Supplementary File/biomolecules-3675083-Western blots/Western blots/Protein molecular weight.pdf]

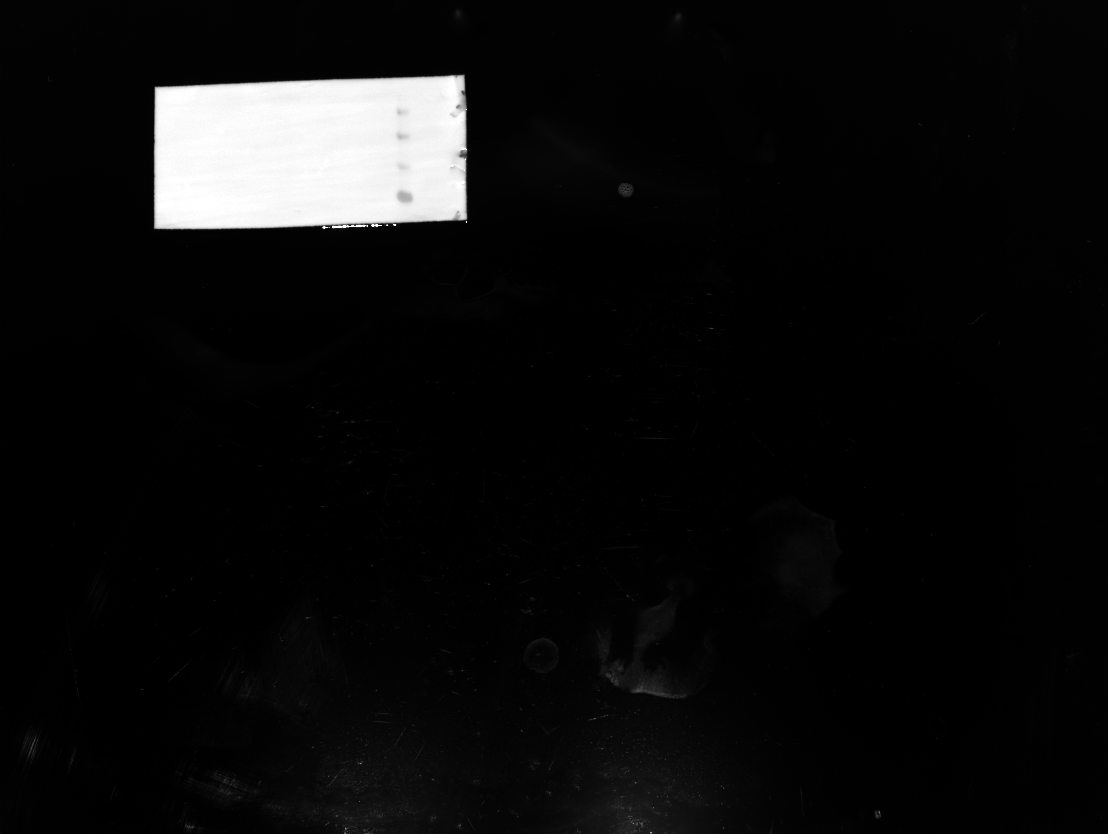

Supplement: Supplementary file 1 [file biomolecules-15-01080-s001.zip › biomolecules-3675083-Supplementary File/biomolecules-3675083-Western blots/Western blots/β-Actin marker.png]

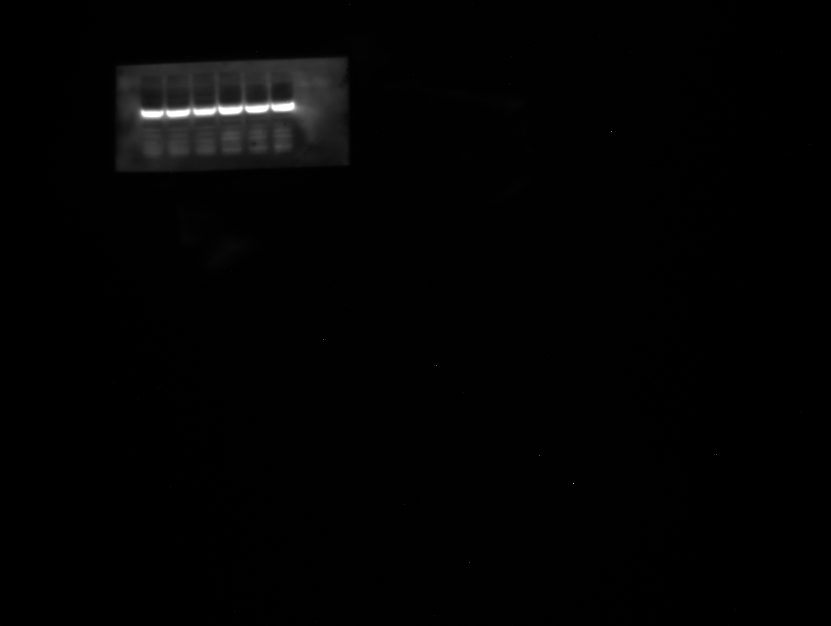

Supplement: Supplementary file 1 [file biomolecules-15-01080-s001.zip › biomolecules-3675083-Supplementary File/biomolecules-3675083-Western blots/Western blots/β-Actin.png]
